# Propolis Prevents Hepatorenal Injury Induced by Chronic Exposure to Carbon Tetrachloride

**DOI:** 10.1155/2012/235358

**Published:** 2011-08-04

**Authors:** Monika Bhadauria

**Affiliations:** School of Studies in Zoology, Jiwaji University, Gwalior, 474011, India

## Abstract

Carbon tetrachloride (CCl_4_) is a well-known hepatotoxicant, and its exposure induces hepatorenal injury via oxidative stress and biochemical alterations. This study had been conducted to confirm the protective role of propolis extract on CCl_4_-induced hepatorenal oxidative stress and resultant injury. Propolis extracts collected from Gwalior district and 24 female Sprague Dawley rats were used for experiment. Animals were exposed to CCl_4_ (0.15 mL/kg, i.p.) for 12 weeks (5 days/week) followed by treatment with propolis extract (200 mg/kg, p.o.) for consecutive 2 weeks. CCl_4_ exposure significantly depleted blood sugar and hemoglobin level and raised the level of transaminases, alkaline phosphatase, lactate dehydrogenase, protein, urea, albumin, bilirubin, creatinine, triglycerides, and cholesterol in serum. Lipid peroxidation was enhanced, whereas GSH was decreased significantly in liver and kidney in CCl_4_-intoxicated group. Ethanolic extract of propolis successfully prevented these alterations in experimental animals. Activities of catalase, adenosine triphosphatase, glucose-6-phosphatase, acid, and alkaline phosphatase were also maintained towards normal with propolis therapy. Light microscopical studies showed considerable protection in liver and kidney with propolis treatment, thus, substantiated biochemical observations. This study confirmed hepatoprotective potential of propolis extract against chronic injury induced by CCl_4_ by regulating antioxidative defense activities.

## 1. Introduction

The chronic liver diseases are common worldwide and are characterized by a progressive evolution from steatosis to chronic hepatitis, fibrosis, cirrhosis, and hepatocellular carcinoma [1, 2]. Free radicals and reactive oxygen species (ROS) play a crucial role in development of liver diseases [3]. The liver is exposed to absorbed drugs or xenobiotics in concentrated form due to its unique vascular and metabolic features. Drug-metabolizing enzymes detoxify many xenobiotics but bioactivate the toxicity of others [4]. In case of bioactivation, liver is the first organ exposed to the damaging effects of newly formed toxic substance. Therefore, protective armaments for liver are of particular interest. Considerable efforts are being made to obtain useful herbal medicines from documented medicinal plants [5] for a wide variety of clinical conditions. Dietary antioxidants of natural products may serve as therapeutics to cope with liver damage [3] against free radicals and ROS-induced liver diseases pathology and progression. Natural antioxidants in complex mixtures if ingested with the diet are more efficacious than pure compounds in preventing oxidative stress-related pathologies due to particular interactions and synergisms [3] by modulating antioxidant, drug-metabolizing, and repairing enzymes along with acting as signaling molecules in important cascades for cell survival [6, 7]. 

Propolis is an adhesive, resinous substance collected, transformed and used by honeybees to seal holes in their honeycombs, smooth out the internal walls, and protect the entrance of intruders. Honeybees collect the resin from cracks in the bark of trees and leaf buds. They bring propolis back to the hive, where it is modified and mixed with other substances, including bees' own wax and salivary secretions [8]. Propolis has been used in folk medicine all over the world. It has anti-inflammatory, immunoregulatory, bacteriostatic, and antibacterial activities [9–11]. It provides excellent cartilage protection suggesting a potential application in joint disease [12]. Long-term administration of propolis does not induce any significant change in seric parameters; thus, it might not have any cardiac injury [13]. Propolis extracts present low toxicity to experimental animals, with LD_50_ higher than 7 g/kg for mice [14, 15]. Previously we reported strong hepatoprotective effect of propolis against acute hepatic damage [16–21] and subchronic hepatic injury induced by CCl_4_ [22] and acetaminophen [23]. In the present communication, an attempt has been made to explore potential of propolis in preventing chronic hepatorenal injury. 

## 2. Material and Methods

### 2.1. Animals and Hepatorenal Injury by CCl_4_

Female Sprague Dawley rats (130 ± 10 g) were kept (3/cage: 6/group) in the animal house under standard husbandry conditions (temp. 25 ± 2°C; relative humidity 60 ± 5%; 12 h light/dark cycle). The animals were fed on pelleted diet (Pranav Agro Industries Ltd., India) and drinking water *ad libitum*. Experiments were conducted in accordance with the guidelines set by the Institutional Animal Ethics Committee (501/01/A/CPCSEA) of Jiwaji University. Hepatorenal injury was induced by CCl_4_ (0.15 mL/kg; diluted with liquid paraffin) that was administered for 12 weeks (5 days/week) [24].

### 2.2. Preparation and Administration of Propolis

Crude propolis from the hive of *Apis mellifera* was collected from apiaries nearby Gwalior district (MP) by Professor O. P. Agrawal, Senior Entomologist, School of Studies in Zoology, Jiwaji University, Gwalior (India). Propolis is a plant-derived product, and it has been proved that bees do not change its chemical composition [25]. It is reported that more than 300 compounds of different classes are present in propolis among which more than 100 are common worldwide. A lot of knowledge has already been gathered on active components, and one of the most important active principles was found to be caffeic acid phenethyl ester [26]. A 90% ethanolic extract was obtained as described previously [16, 17], yield of dried residue was about 61.4% (w/w) and kept at 4°C for further use. Aqueous suspension of propolis was prepared in 1% gum acacia suspension, and selected optimum dose was administered (200 mg/kg, p.o.) to the animals for 2 weeks on the basis of our previous studies [22, 23]. Silymarin was given as positive control in respect to propolis.

### 2.3. Experimental Design

Animals of group 1 received vehicle only and served as normal control. Animals of group 2–4 received CCl_4_ for 12 weeks (5 days/week) and group 2 served as experimental control. Group 3 and 4 received treatments of propolis and silymarin, respectively, for 2 weeks (5 days/week). Blood was collected from retro-orbital venous plexus after 48 h of the last administration. The animals of entire groups were euthanized; liver and kidney were immediately excised and processed for biochemical analyses and histological preparations. Scheme of different treatments is given in Table 1.

### 2.4. Isolation of Serum and Homogenate Preparation

Serum was isolated after keeping the blood for 1 h at room temperature followed by centrifugation at 1000 g for 15 min and stored at −20°C until analyzed. Tissue samples of liver and kidney were homogenized with ice-cold 150 mM KCl for the determination of TBARS and CAT activity and in 1% sucrose for GSH determination. Homogenates of liver and kidney were prepared in chilled hypotonic solution (10% w/v) for other biochemical assays, that is, total protein, cholesterol, adenosine triphosphatase (ATPase), acid, and alkaline phosphatase (ACPase and ALPase). 

### 2.5. Determination of Hepatorenal Marker Enzymes in Serum and Other Blood Biochemical Endpoints

Blood was immediately used for determination of hemoglobin [27] and blood sugar level [28]. Serum was used for the determination of aspartate aminotransferase (AST) [29], alanine aminotransferase (ALT) [29], alkaline phosphatase (SALP) [30], lactate dehydrogenase (LDH) [31], and serum protein contents [32]. Serum bilirubin, albumin, urea, creatinine, and triglycerides were determined by E-Merck's kit according to the manufacturer's instructions. 

### 2.6. Assessment of Peroxidative Stress and Antioxidant Status

Lipid peroxidation (LPO) was measured using thiobarbituric acid (TBA) [33] and expressed as *n* moles TBARS/g tissue using an extinction coefficient of 1.56 × 105/M/cm. The GSH measurement was performed using dithionitrobenzoic acid [34]. The GSH level was calculated using an extinction coefficient of 13600/M/cm and expressed as *μ* moles GSH/g tissue. Catalase activity was determined as per method of Aebi [35]. Decomposition of H_2_O_2_ was monitored by decrease in the absorbance at *λ* 240 nm. H_2_O_2_ concentration was calculated using extinction coefficient of 0.0394/mM/cm and activity was expressed as *n* moles H_2_O_2_/min/mg protein.

### 2.7. Tissue Biochemical Assay

Fresh tissues of liver and kidney were immediately processed to determine glycogen by anthrone reagent method [36]. Total proteins were determined using bovine serum albumin as standard, and blue color was developed by the reaction of proteins and Folin-Ciocalteau reagent [32]. Hepatic and renal triglycerides were determined according to Neri and Frings [37]. Determination of enzymatic activities including ACPase [30], ALPase [30], and ATPase [38] was also performed in liver and kidney. 

### 2.8. Histopathological Observations

For histological studies, samples from liver and kidney were fixed in Bouin's fixative and processed routinely for embedding in paraffin. Tissue sections of 5 *μ*m thickness were stained with hematoxylin and eosin (H&E) and examined under compound light microscope.

### 2.9. Statistics

Data are expressed as mean ± SE of six animals used in each group. Statistical analysis was carried out by one way analysis of variance (ANOVA) considered significant at *P* ≤ 0.05 followed by Student's *t*-test [39]. 

## 3. Results

### 3.1. Hepatorenal Marker Enzymes in Serum and Other Blood Biochemical Endpoints

**Figure 1:**
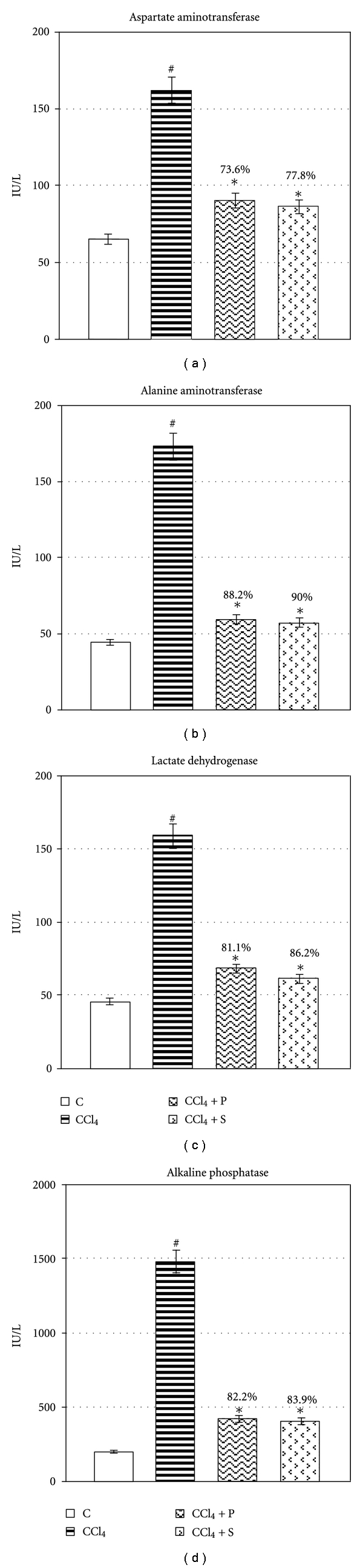
Showing therapeutic potential of propolis on CCl_4_-induced alteration in liver marker enzymes of serum. Values are mean ± SE of *n* = 6 in each group. @Significant for ANOVA at 5% level. *P* value CCl_4_ versus normal at #≤0.05; *P* value treatment versus CCl_4_ at ∗ ≤ 0.05 for Student's *t*-test; *F* variance for ANOVA of AST = 60.3^†^, ALT = 145^†^, LDH = 114^†^, SALP = 204^†^. Abbreviations: Control (C); Carbon tetrachloride (CCl_4_); Propolis (P); Silymarin (S); Aspartate aminotransferase (AST); Alanine aminotransferase (ALT); Lactate dehydrogenase (LDH), and Alkaline phosphatase (SALP).

**Figure 2:**
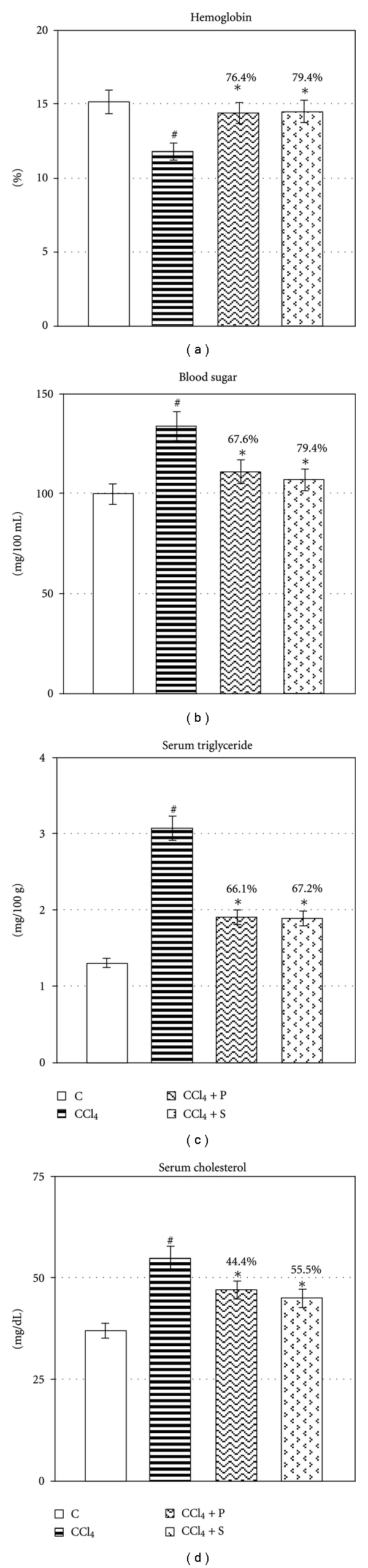
Showing therapeutic potential of propolis on CCl_4_-induced alteration in serological parameters. Values are mean ± SE of *n* = 6 in each group. @Significant for ANOVA at 5% level. *P* value CCl_4_ versus normal at #≤0.05; *P* value treatment versus CCl_4_ at ∗ ≤ 0.05 for Student's *t*-test; *F* variance for ANOVA of hemoglobin = 4.44^†^, blood sugar = 6.57^†^, triglycerides = 46.9^†^, and cholesterol = 7.81^†^. Abbreviations: Control (C); Carbon tetrachloride (CCl_4_); Propolis (P); Silymarin (S).

**Figure 3:**
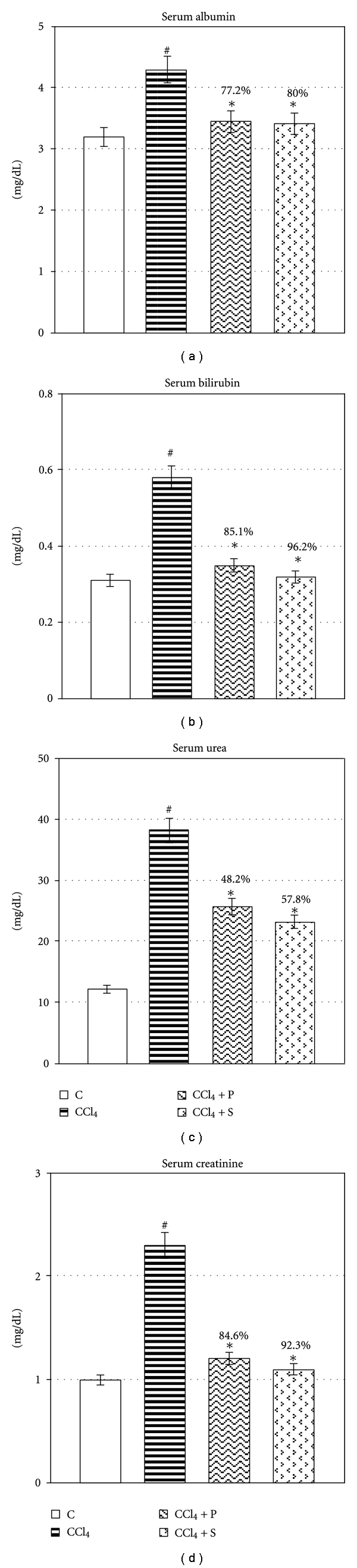
Showing therapeutic potential of propolis on CCl_4_-induced alteration in specific serum markers. Values are mean ± SE of *n* = 6 in each group. @Significant for ANOVA at 5% level. *P* value CCl_4_ versus normal at #≤0.05; *P* value treatment versus CCl_4_ at ∗ ≤ 0.05 for Student's *t*-test; *F* variance for ANOVA of albumin = 7.34^†^, bilirubin = 39.0^†^, urea = 64.1^†^, and creatinine = 56.1^†^. Abbreviations: Control (C); Carbon tetrachloride (CCl_4_); Propolis (P); Silymarin (S).

Chronic exposure to CCl_4_ exhibited elevation in the leakage of AST, ALT, LDH, and SALP significantly (*P* ≤ 0.05) (Figure 1). Treatment of propolis extract significantly reduced the leakage of AST, ALT, LDH, and SALP in circulation (*P* ≤ 0.05), thereby, confirming its protective effect in chronic injury. Chronic administration of CCl_4_ decreased hemoglobin level, whereas significant rise was observed in blood sugar, serum triglycerides and cholesterol (*P* ≤ 0.05) (Figure 2). Propolis therapy significantly reversed the level of hemoglobin and blood sugar as well as serum triglycerides and cholesterol towards control (*P* ≤ 0.05). CCl_4_ exposure significantly increased serum albumin, bilirubin, urea, and creatinine (*P* ≤ 0.05) (Figure 3). Treatment with propolis for 2 weeks significantly decreased the level of these serological variables and brought the values very near to control group (*P* ≤ 0.05). Efficacy of propolis was well compared with positive control silymarin, and percent protection clearly showed that propolis possesses almost equal degree of protection in all biochemical endpoints as silymarin-treated positive control.

### 3.2. Peroxidative Stress and Antioxidant Status

**Figure 4:**
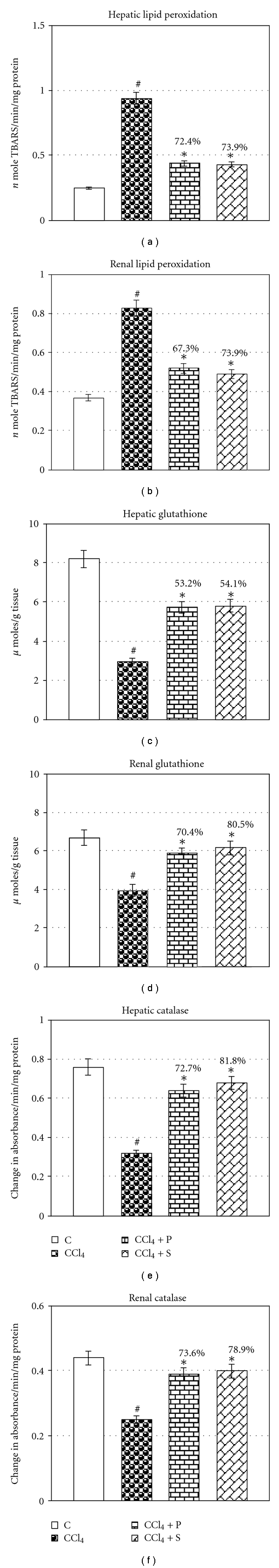
Showing therapeutic potential of propolis on CCl_4_-induced hepatorenal oxidative stress. Values are mean ± SE of *n* = 6 in each group. @Significant for ANOVA at 5% level. *P* value CCl_4_ versus normal at #≤0.05; *P* value treatment versus CCl_4_ at ∗ ≤ 0.05 for Student's *t*-test; *F* variance for ANOVA of hepatic LPO = 104^†^, renal LPO = 45.2^†^, hepatic GSH = 50.3^†^, renal GSH = 27.7^†^, hepatic CAT = 37.7^†^, and renal CAT = 18.9^†^. Abbreviations: Control (C); Carbon tetrachloride (CCl_4_); Propolis (P); Silymarin (S).

Peroxidative stress and antioxidant status both in liver and kidney tissues were determined in terms of LPO, GSH, and catalase (Figures 4(a)–4(f)). CCl_4_ administration enhanced the formation of TBARS in hepatic and renal tissues after its chronic exposure (*P* ≤ 0.05). Therapy with propolis extract diminished the production of TBARS (*P* ≤ 0.05), which ultimately reduced peroxidative stress up-to a considerable extent. The level of GSH and catalase was significantly decreased in liver and kidney after administration of CCl_4_ (*P* ≤ 0.05). Propolis extract was therapeutically effective in restoring GSH level and in maintaining CAT enzyme in liver and kidney in a significant manner (*P* ≤ 0.05), which could help in mitigating oxidative stress.

### 3.3. Tissue Biochemical Assay

Significantly enhanced enzymatic activity of ACPase was found in liver and kidney after CCl_4_ exposure (*P* ≤ 0.05; Table 2). Propolis therapy was significantly effective in reducing elevated activity of ACPase in both tissues (*P* ≤ 0.05). Significant fall was noticed in activities of ALPase and ATPase in liver and kidney after 12-week administration of CCl_4_ (Table 2; *P* ≤ 0.05). Therapeutic effect of propolis extract was found to be significant in both organs, and altered enzymatic activities were reversed towards control (*P* ≤ 0.05). Significant fall in total protein and glycogen contents was observed after chronic exposure to CCl_4_, whereas triglycerides were increased significantly in liver and kidney (*P* ≤ 0.05; Table 3). Therapy with propolis extract alleviated total protein and glycogen contents and regulated hepatic triglycerides towards control (*P* ≤ 0.05). Propolis was found to be equally effective as ilymarin on the basis of percent protection.

### 3.4. Histopathological Observations

**Figure 5:**
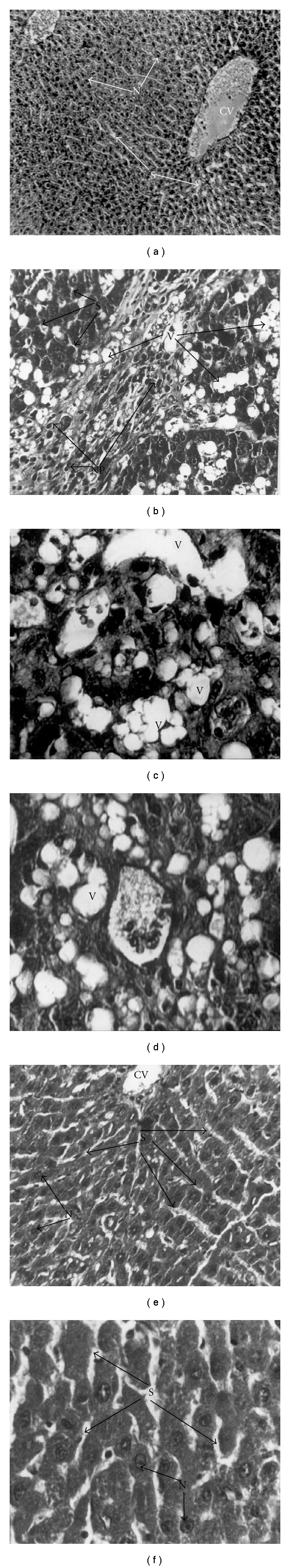
(a) Photomicrograph of control liver with polygonal hepatocytes cordially arranged around hepatic central vein. Hepatic nuclei (N), uniform sinusoidal spaces (S) terminating into central vein (CV) were clearly visible (200x). (b) Liver sections of CCl_4_ induced chronic hepatic injury with disturbed cord arrangement of hepatocytes, vacuolation (V) due to collapsed cellular membranes, high accumulation of nonparenchymal or inflammatory cells (NP) and irregular sinusoids (200x). (c and d) Liver sections of CCl_4_ induced chronic hepatic injury with disturbed cord arrangement of hepatocytes and high degree of vacuolation (V) due to collapsed cellular membranes (400x). (e) Photomicrograph of liver after propolis therapy with minimal degree of vacuolation, better formed polygonal hepatocytes cordially arranged towards hepatic central vein (CV). Hepatic nuclei (N), uniform sinusoids (S) terminating into central vein were clearly visible (200x). (f) Silymarin therapy with better formed features of liver histology as comparable to experimental control (400x).

Liver of control rats depicted regular histoarchitecture (Figure 5(a)). Liver sections of chronic CCl_4_ toxicity showed degeneration so liver cells were seen swollen. It is encountered simultaneously with ballooning degeneration and steatosis. Focal necrosis was seen clearly. Nuclear changes such as karyopyknosis and degeneration of the cell membrane indicated necrosis. These liver showed presence of fibrous septa with heterogenous population of nonparenchymal cells, foamy degeneration due to plenty of vacuolation and disturbed hepatic cord array (Figures 5(b), 5(c), and 5(d)). The protective effect of propolis against CCl_4_-induced chronic liver damage and cirrhosis was confirmed by conventional histological examinations. Liver sections obtained from animals, those followed by propolis therapy, showed consistent reduction of liver necrosis and inflammation. Moreover, the cirrhosis process was seen reduced. Proper sinusoidal spaces and cord arrangement were clearly visible with binuclear hepatocytes indicating regenerative effects (Figure 5(e)). Silymarin treatment also improved histoarchitecture showing well-formed hepatic cord arrangements with clearly visible nuclei and sinusoidal spaces (Figure 5(f)). 

**Figure 6:**
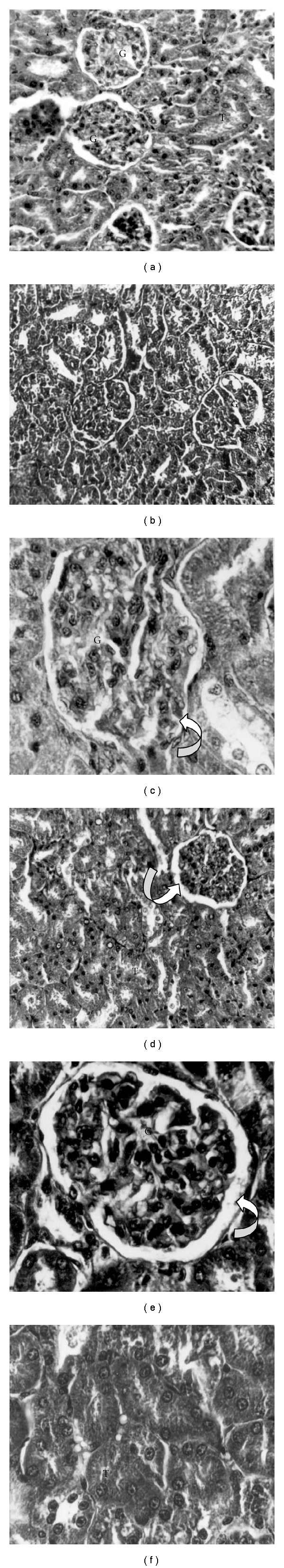
(a) Photomicrograph of kidney of control rat showing well-formed Bowman's capsule and normal glomeruli (G), uniform space between glomerulus and capsule wall, tubular lumen with basal and apical nuclei (200x). (b) CCl_4_ administration caused swelling in glomerulus and tubular obstruction. loss of glomerulus space (thick arrows) and (200x). (c) CCl_4_ exposure caused swelling in glomerulus so that space between glomerulus and capsule wall (curved arrow) was reduced (400x). (d): Administration of propolis retained the kidney histoarchitecture with opened lumen of tubules (T) and uniform space between glomerulus and capsule wall (curved arrow) (200x). (e) Propolis therapy maintained uniform space between glomerulus and capsule wall (curved arrow) (400x). (f) Silymarin therapy recovered the kidney histoarchitecture with wider lumen of tubules (T) (400x).

Kidney of control rat showed regular histological features (Figure 6(a)). Administration of CCl_4_ for 12 weeks provoked histopathological lesions, including disruption in the epithelial cells of the tubules; proximal tubules showed hypertrophy in proximal tubules with debris in the lumen due to which the lumen was found to be obstructed. The glomeruli occupied whole Bowman's capsule, medullary tubules showed degeneration, and nuclei showed apical position (Figures 6(b) and 6(c)). Propolis therapy made proximal tubules well organized (Figure 6(d)), glomeruli reversed to the regular shape leaving wider space between glomerulus and Bowman's capsule wall (Figure 6(e)). Administration of silymarin improved histological features of kidney. Endothelial lining was improved, and cortical and medullary tubules were found to be well formed (Figure 6(f)).

## 4. Discussion

Various environmental toxicants and clinically useful drugs can cause severe cellular damages in different organs through their metabolic activation. CCl_4_ is one of such extensively studied toxicants that has been used to induce liver injury for evaluation and confirmation of hepatoprotective drugs [40]. The reactive metabolite trichloromethyl radicals (^•^CCl_3_) are formed from the metabolic conversion of CCl_4_ by cytochrome P-450 [41]. As O_2_ tension rises, greater fraction of ^•^CCl_3_ present in the system reacts rapidly with O_2_, and many orders of magnitude of more reactive free radicals CCl_3_OO^•^ are generated [42]. These free radicals initiate peroxidation of membrane polyunsaturated fatty acids [43] and covalently bind to microsomal lipids and proteins [44] resulting in ROS. Various enzymatic and nonenzymatic systems have been developed by the cell to cope with ROS and other free radicals. However, a condition of oxidative stress establishes due to insufficient defense capacities against ROS [45]. The ROS also affect antioxidant defense mechanisms, reduce intracellular concentration of GSH, decrease the activity of CAT, and cause organ injury and carcinogenesis [46].

Lipid peroxidations as well as altered levels of some endogenous scavengers are taken as reliable indices for oxidative stress [47, 48]. In the present study, administration of CCl_4_ enhanced LPO in liver and kidney. Treatment of propolis extract inhibited the generation of TBARS in both tissues that confirmed its antilipid peroxidative effects in chronic condition of hepatorenal injury. GSH constitutes the first line of defense against free radicals [18]. Another defense mechanism involves antioxidant enzymes, including CAT that converts active oxygen molecules into nontoxic compounds. CCl_4_ administration decreased the activity of CAT and reduced GSH concentration in the tissues, which is in agreement with earlier reports [19, 20]. Oral administration of propolis reactivated the activities of CAT and restored GSH level, which in turn increased the detoxification of active metabolites of CCl_4_. Flavonoids and their esters in propolis are pharmacologically active molecules and have been hypothesized to influence the antioxidant activity of propolis [49, 50]. AST, ALT, SALP, and LDH are sensitive markers of liver injury, and several fold increase in the release of these enzymes indicated severity of damage in chronic study. Serum bilirubin, albumin, urea, creatinine, and triglycerides were also found to be abnormally higher, which indicated hepatic and renal damage. Administration of propolis extract brought these endpoints towards control indicating improvement in metabolic processes. In most of the parameters, efficacy of propolis was found to be the same as sylimarin treatment. Evidently, histopathological examinations of liver and kidney also supported propolis therapy as it helped in improving cellular architecture. This clearly indicated membrane stabilizing effect of propolis probably by scavenging free radicals. 

GSH depletion increases the sensitivity of cells to various aggressions and also has several metabolic effects, for example, a decrease in the rate of gluconeogenesis or an increase in glycogenolysis [51]. Fall in protein and glycogen contents in liver and kidney tissues was observed in this investigation that might be associated with increased hepatorenal injury, which in turn resulted into decreased capacity of synthesize protein and glycogen. Propolis therapy augmented protein and glycogen contents in liver and kidney, which indicated that propolis prevented hepatorenal injury and improved physiology of these organs by modulating cellular metabolism and regeneration.

Activities of membranes enzymes ATPase and ALPase were decreased considerably in liver and kidney after CCl_4_ exposure [19, 23, 24]. Free radicals are produced inside mitochondria and are frequently released into the cytosol. The production of ATP in the mitochondria involves the transport of protons across the inner mitochondrial membrane via the electron transport chain. Uncoupling of oxidative phosphorylation leads to fall in activity of ATPase. The use of ATPase level measurement was considered as an appropriate index of membrane damage. Significant recovery was found in these enzymes with the treatment of propolis. It may be suggested that impaired mitochondrial oxidative phosphorylation in liver is retained thereby preventing depletion of ATP energy stores [5]. Considerable reversal in ATPase, ALPase, and ACPase activities indicated membrane stabilizing effect of propolis extract. Propolis is also supposed to be helpful in absorption and utilization of various minerals due to the presence of organic acid derivatives in it, which in turn improved physiological functions by regulating the ion dependent enzymatic activities [52]. Efficacy of propolis was found to be the same as of silymarin on the basis of percent protection.

Since, liver and kidneys are closely related organs, which take part in different metabolic and excretion events. Any abnormality in liver may also lead to impairment in kidney functions. That is why both the organs had been taken into consideration in this study. In conclusion, results of this study validate the folklore use of propolis against various ailments. Propolis is interestingly effective in ameliorating acute, subchronic, and chronic injury to liver. It also has wider therapeutic index, and thus it may serve as clinically useful hepatoprotective natural product in future. 

## Figures and Tables

**Table 1:** Scheme for different treatments and durations.

Treatments	1st–12th weeks	13th-14th weeks	Last day of 14th week
Gr 1: normal control	Liquid paraffin	Gum acacia suspension	Euthanized
Gr 2: experimental control	CCl_4_ (0.15 mL/kg)	Gum acacia suspension	Euthanized
Gr 3: propolis treatment	CCl_4_ (0.15 mL/kg)	Propolis (200 mg/kg)	Euthanized
Gr 4: silymarin treatment	CCl_4_ (0.15 mL/kg)	Silymarin (50 mg/kg)	Euthanized

**Table 2:** Potential of propolis in maintaining metabolizing enzymes. (Values are mean ± SE from six rats in each group.)

Groups	Adenosine triphosphatase		Acid phosphatase		Alkaline phosphatase	
	(mg Pi/100 mg/min)		(mg Pi/100 mg/h)		(mg Pi/100 mg/h)	
	Hepatic	Renal	Hepatic	Renal	Hepatic	Renal
Control	2020 ± 111	2500 ± 138	250 ± 30.9	270 ± 14.9	74.0 ± 4.09	2580 ± 142
CCl_4_	987 ± 54.5^#^	1095 ± 60.5^#^	1395 ± 77.1^#^	1440 ± 79.6^#^	36.0 ± 1.99^#^	716 ± 39.6^#^
CCl_4_ + Propolis	1742 ± 96.2*	1775 ± 98.1*	338 ± 18.7*	378 ± 20.9*	68.0 ± 3.75*	1591 ± 87.9*
*% Protection*	*73.1%*	*48.4%*	*92.3%*	*90.7%*	*84.2%*	*46.9%*
CCl_4_ + Silymarin	1806 ± 99.8*	1880 ± 232*	335 ± 18.5*	342 ± 18.9*	69.0 ± 3.81*	1665 ± 92.1*
*% Protection*	*79.3%*	*55.8%*	*92.6%*	*93.8%*	*86.8%*	*50.9%*

*F* Variance	28.1^†^	36.7^†^	208^†^	202^†^	29.3^†^	73.1^†^

*P* value CCl_4_ versus control at #≤0.05; therapy versus CCl_4_ at ∗ ≤ 0.05; ^†^significant for analysis of variance *P* ≤ 0.05.

**Table 3:** Effect of propolis on hepatorenal biochemical assay against carbon tetrachloride intoxication. (Values are mean ± SE from six rats in each group.)

Groups	Total protein contents		Glycogen contents		Triglycerides	
	(mg/100 mg)		(mg/100 g)		(mg/100 mg)	
	Hepatic	Renal	Hepatic	Renal	Hepatic	Renal
Control	15.7 ± 0.86	15.1 ± 0.83	2700 ± 149	84.0 ± 4.64	3.20 ± 0.17	2.95 ± 0.16
CCl_4_	12.3 ± 0.67^#^	13.1 ± 0.72^#^	984 ± 54.3^#^	51.1 ± 2.81^#^	8.30 ± 0.45^#^	6.78 ± 0.37^#^
CCl_4_ + Propolis	15.3 ± 0.84*	14.1 ± 0.77*	2128 ± 117*	79.0 ± 4.36*	3.80 ± 0.21*	3.37 ± 0.18*
*% Protection*	*88.2%*	*50.0%*	*66.6%*	*84.8%*	*88.2%*	*89.1%*
CCl_4_ + Silymarin	15.4 ± 0.85*	14.3 ± 0.79*	2240 ± 123*	81.0 ± 4.47*	3.60 ± 0.19*	3.58 ± 0.19*
*% Protection*	*91.1%*	*60.0%*	*73.1%*	*90.9%*	*92.2%*	*83.5%*

*F* Variance	4.58^†^	1.32	46.9^†^	163^†^	84.6^†^	61.6^†^

*P* value CCl_4_ versus control at #≤0.05; therapy versus CCl_4_ at ∗ ≤ 0.05; ^†^significant for analysis of variance *P* ≤ 0.05.
